# A robust Pax7EGFP mouse that enables the visualization of dynamic behaviors of muscle stem cells

**DOI:** 10.1186/s13395-018-0169-7

**Published:** 2018-08-24

**Authors:** Elisia D. Tichy, David K. Sidibe, Christopher D. Greer, Nicholas M. Oyster, Panteleimon Rompolas, Nadia A. Rosenthal, Helen M. Blau, Foteini Mourkioti

**Affiliations:** 1 Department of Orthopaedic Surgery, Perelman School of Medicine, The University of Pennsylvania, Philadelphia, PA USA; 2 Cell and Molecular Biology Graduate Program, The University of Pennsylvania, Philadelphia, PA USA; 3 Department of Dermatology, Institute for Regenerative Medicine, Perelman School of Medicine, University of Pennsylvania, Philadelphia, PA USA; 4 The Jackson Laboratory, Bar Harbor, ME USA; 5 Sackler School of Graduate Biomedical Sciences, Tufts University, Boston, MA USA; 6 The Jackson Laboratory for Genomic Medicine, Farmington, CT USA; 7 Australian Regenerative Medicine Institute, Monash University, Melbourne, VIC Australia; 8 National Heart and Lung Institute, Imperial College London, London, UK; 9 Baxter Laboratory for Stem Cell Biology, Department of Microbiology and Immunology, Institute for Stem Cell Biology and Regenerative Medicine, Stanford School of Medicine, Stanford, CA USA; 10 Department of Cell and Developmental Biology, Penn Institute of Regenerative Medicine, Musculoskeletal Regeneration Program, Perelman School of Medicine, The University of Pennsylvania, Philadelphia, PA USA; 11 Musculoskeletal Regeneration Program, Department of Orthopaedic Surgery and Cell and Developmental Biology, Penn Institute of Regenerative Medicine, The University of Pennsylvania, 3450 Hamilton Walk, 112A Stemmler Hall, Philadelphia, PA 19104-6081 USA

**Keywords:** Skeletal muscle, Pax7, EGFP, Muscle stem cell, Satellite cell, Muscle development, Muscle regeneration, Fluorescence-activated cell sorting

## Abstract

**Background:**

Pax7 is a transcription factor involved in the specification and maintenance of muscle stem cells (MuSCs). Upon injury, MuSCs leave their quiescent state, downregulate Pax7 and differentiate, contributing to skeletal muscle regeneration. In the majority of regeneration studies, MuSCs are isolated by fluorescence-activated sorting (FACS), based on cell surface markers. It is known that MuSCs are a heterogeneous population and only a small percentage of isolated cells are true stem cells that are able to self-renew. A strong Pax7 reporter line would be valuable to study the in vivo behavior of Pax7-expressing stem cells.

**Methods:**

We generated and characterized the muscle properties of a new transgenic Pax7EGFP mouse. Utilizing traditional immunofluorescence assays, we analyzed whole embryos and muscle sections by fluorescence microscopy, in addition to whole skeletal muscles by 2-photon microscopy, to detect the specificity of EGFP expression. Skeletal muscles from Pax7EGFP mice were also evaluated in steady state and under injury conditions. Finally, MuSCs-derived from Pax7EGFP and control mice were sorted and analyzed by FACS and their myogenic activity was comparatively examined.

**Results:**

Our studies provide a new Pax7 reporter line with robust EGFP expression, detectable by both flow cytometry and fluorescence microscopy. Pax7EGFP-derived MuSCs have identical properties to that of wild-type MuSCs, both in vitro and in vivo*,* excluding any positional effect due to the transgene insertion. Furthermore, we demonstrated high specificity of EGFP to label MuSCs in a temporal manner that recapitulates the reported Pax7 expression pattern. Interestingly, immunofluorescence analysis showed that the robust expression of EGFP marks cells in the satellite cell position of adult muscles in fixed and live tissues.

**Conclusions:**

This mouse could be an invaluable tool for the study of a variety of questions related to MuSC biology, including but not limited to population heterogeneity, polarity, aging, regeneration, and motility, either by itself or in combination with mice harboring additional genetic alterations.

**Electronic supplementary material:**

The online version of this article (10.1186/s13395-018-0169-7) contains supplementary material, which is available to authorized users.

## Background

The Pax7 transcription factor is a component of the paired box family, which is involved in the specification and maintenance of stem and progenitor cells [1] across multiple species [2–7], including humans [8]. During early murine embryonic development, beginning around e8.5, Pax7 is expressed in neural crest cells, the neural tube, the frontonasal region, and the somites [9, 10], which ultimately form skeletal muscle [11]. As development progresses, the activity of Pax7 contributes to bones of the skull, the brain, olfactory epithelium, nasal septum cartilage, as well as skeletal muscle, in conjunction with Pax3 [9, 10, 12].

From the original characterization of Pax7 [2] as a marker of satellite cells (also called muscle stem cells or MuSCs), there has been a continuous effort among scientists to understand its function. Ablation of Pax7 at conception resulted in a substantial loss of satellite cells in pups, which displayed muscle defects [2, 13, 14]. In non-injured adult muscle, MuSCs remain quiescent, are transcriptionally inactive and express high levels of Pax7 protein [15]. As result of an injury, they become activated; proliferate and differentiate into myogenic progenitors. The activity of the Pax7 transcription factor contributes to MuSC self-renewal, and Pax7^+^ cells can contribute to myogenesis following injury in the adult [15], as evidenced by transplantation models of freshly isolated cells [16].

During the muscle regeneration process, particularly at the proliferation stage of MuSCs, the expression of Pax7 is carefully controlled [17, 18]. Specifically, MuSCs that maintain a high expression of Pax7 protein retain template DNA strands, exhibit lower metabolic activity, and express stem cell markers, while MuSCs exhibiting low Pax7 expression undergo random DNA segregation and are myogenically committed during the regeneration process [19]. MuSCs studies remain dependent on prospective cell isolation methods using fluorescence-activated cell sorting (FACS). Since most of the cell surface markers used in flow cytometry are also expressed on other cell types, these markers can only be utilized in combinations and together with lineage negative selections [16, 20–23]. Recent evidence demonstrates that MuSC population is heterogeneous [20, 24–30]. They differ in their gene expression signatures, differentiation, lineage potential, and stemness, since only a small percentage of isolated cells are true stem cells able to self-renew (discussed in [18]). Using single fiber isolation methods, muscle stem cells have been shown to self-renew through asymmetric division to refill the stem cell reservoir and to produce more committed progenitors, which are associated with muscle growth and regeneration [31]. Implementing Cre-Lox P-based lineage-tracking methods to analyze the functional role of Pax7 in adult muscle stem cells resulted in different outcomes [32–34], presumably due to limitations in Cre-Lox technology and the contribution of stem cells that escaped gene recombination (discussed in [35]).

Deciphering the function(s) of Pax7 in an in vivo context has proven difficult. In this study, we describe and analyze the properties of a new transgenic reporter mouse model, Pax7EGFP, which marks the muscle stem cells, which are both Pax7 and CD34/α7-integrin dual-positive. Importantly, we have found that the EGFP protein in our mouse is a dynamic marker of Pax7, which gradually declines upon MuSC activation. We further show that the EGFP intensity of Pax7EGFP MuSCs is very bright and can be visualized by fluorescence microscopy both in vitro (native and antibody stained) but more importantly in vivo (by two-photon microscopy), making this mouse model an excellent tool to study the dynamic behaviors of MuSCs in live animals during homeostasis and/or disease conditions.

## Methods

### Pax7EGFP mouse generation

The EGFP coding sequence was inserted in-frame immediately downstream of the first exon of Pax7, within the RP23-204F20 bacterial artificial chromosome (BAC; start position: 82608). This 213 Kb BAC contains DNA sequence 81 Kb upstream to 34 Kb downstream of the *Pax7* locus. Thus, the *Pax7* endogenous promoter and regulatory elements drive expression of the EGFP. The resulting construct, named *Pax7-EGFP* hereafter, was linearized and microinjected into the pronuclei of fertilized eggs, which were then implanted into pseudopregnant female mice. Progeny were analyzed for genomic integration of the transgene by PCR. Transgene-positive progeny (founders) were crossed with wild-type C57Bl6 mice (Stock #000664 from Jackson Laboratories) to facilitate the expansion of the lines. MuSCs were isolated from mice deriving from these lines and were further screened for the expression level of EGFP protein by flow cytometry. The line with the most robust expression of EGFP in MuSCs (Additional file 1: Figure S1) was amplified further to establish the Pax7EGFP line.

### Experimental mice

The Pax7EGFP heterozygous mice were compared to wild-type mice or control Pax7EGFP negative littermates. For some experiments (Additional files 2 and 3: Figures S2 and S3), Rosa^mTmG^/Pax7Cre heterozygous mice (breeding of Jackson Labs stocks: #007676 and #010530 homozygotes) were also used for comparisons. All mice were housed and bred in accordance with the IACUC guidelines of the University of Pennsylvania.

### Genotyping

To identify which mice carry the Pax7EGFP BAC, genomic DNA was isolated from ear snips with genomic DNA isolation buffer (100 mM Tris, pH 8.0, 5 mM EDTA, 200 mM NaCl, 0.2% SDS, 0.2 mg/mL proteinase K) Primers utilized were P7EGFP-pr1: 5′-TGAAAGGAAGAGACGCCAAG-3′, and P7EGFP-pr2: 5′- TCGTTGGGGTCTTTGCTCAG-3′. PCR products were generated with GoTaq Green (Promega) under the following conditions a 94 °C hold for 2′, 36 cycles of 94 °C for 30″, 56 °C for 30″, 72 °C for 1′ followed by a 72 °C hold for 10′. Mice that are positive for Pax7EGFP (both homozygous and heterozygous) will yield a 706-basepair product.

### Embryo isolation and imaging

To isolate embryos, Pax7EGFP heterozygous male mice were bred with wild-type C57Bl/6 females (stock #000664 from Jackson Labs). Following confirmation of plugs, males were removed from the cage. Pregnant mothers were sacrificed 11.5 or 14.5 days after timed mating set-up. Embryos were dissected from the uterine horns and imaged using an Olympus SZX2 stereomicroscope equipped with fluorescence. Fluorescent and bright field images were merged using Fiji.

### Muscle stem cell (MuSC) isolation

Hindlimb muscles (quadriceps, gastrocnemius, and tibialis anterior) were dissected and MuSCs collected as described [36]. Briefly, tissue was enzymatically dissociated with 0.1% collagenase (Sigma) and 4.8 units/mL dispase (Roche) in DMEM, using the gentleMACs system (Miltenyi Biotech). The cell slurry was pulled through a 21-gauge needle until all remaining muscle tissue was broken apart, after which the cell solution was filtered through a 40 μm cell strainer. Red blood cells were eliminated with red cell lysis buffer (eBioscience). Cells were stained with biotinylated antibodies (CD31 from eBioscience; Sca1, CD45, and CD11b from BD Biosciences) followed by staining with streptavidin-conjugated PE-Cy7 (BioLegend), Alexa Fluor 647-conjugated α7-integrin antibody (AbLab), and Brilliant Violet 421-conjugated CD34 antibody (BD Biosciences). The viability dye 7-AAD (Sigma) was added and cells were either sorted on a FACS Aria II cell sorter (BD Biosciences) or analyzed on an LSRII (BD Biosciences).

### MuSC in vitro expansion and differentiation

MuSCs were FACS-sorted and plated on laminin-coated 8-well chamber slides (Nunc LabTek II; for proliferation) or 96-well collagen-coated plates (for differentiation) in myoblast media [DMEM/F12 supplemented with 15% FBS, 1× Glutamax, 1× antibiotic-antimycotic, and 1/10,000 dilution of 25 μg/mL bFGF (Promega)]. After culturing for 2 days, cells were either fixed in 4%PFA/PBS (for Pax7 and MyoD staining) or were washed and fresh myoblast media was media added. After three more days of culture, cells were transferred to differentiation medium (DMEM, 5% horse serum, 1× Glutamax, 1× antibiotic-antimycotic) for 2 days before fixation with 4% PFA/PBS. Cells were permeabilized in 0.5% Triton X-100/PBS and stained with antibodies against Pax7 (mouse monoclonal; DSHB supernatant; 1/10), MyoD (either mouse monoclonal, DAKO clone 5.8A, 1/50 or rabbit polyclonal, C-20, Santa Cruz 1/50), GFP-tag (ThermoFisher, 1/200), myosin heavy chain (Santa Cruz, clone H300, rabbit polyclonal, 1/50 dilution), and myogenin (DSHB supernatant, clone F5D, 1/10). Secondary antibodies used were Alex Fluor 555 goat anti-mouse IgG, and Alexa Fluor 647 or Alexa Fluor 488 goat anti-rabbit IgG, all at 1/500 dilution. Coverslips were mounted with Fluromount G with DAPI (SouthernBiotech). Pax7, MyoD, and GFP stained cells were imaged on a Nikon eclipse Ni-U equipped with a Nikon Qi1Mc 14-bit camera, while embryonic myosin heavy chain and myogenin were imaged on a Zeiss confocal 710 microscope.

### MuSC live cell imaging

Twenty thousand FACS-sorted GFP^+^ MuSCs from Pax7EGFP heterozygous mice were plated on collagen-coated 16-well chamber slides (Nunc LabTek II) in live-cell imaging medium (DMEM/F12 without phenol red, 15% FBS, 1× Glutamax, 1× antibiotic-antimycotic, 25 μg/mL bFGF). MuSCs were imaged for the presence of GFP 0, 12, 24, 48, 72, and 96 h after plating with a Nikon Eclipse TE2000-U inverted microscope equipped with a Plan Fluor ELWD 20× Ph1 ADL objective and a Pco.edge sCMOS 16-bit camera. All imaging for GFP was taken at the same exposure/intensity settings, where multiple random fields (*N* = 3) were imaged per well at each time point. *n* = 3 mice, where the MuSCs from each mouse were plated in their own well.

### Muscle fiber histology and analysis

Non-injured gastrocnemius, quadriceps, and tibialis anterior muscles were isolated from Pax7EGFP heterozygous mice or control (Pax7EGFP negative) mice of the same gender. Muscles were fixed in 2% PFA/PBS for 2 h at 4 °C and placed in 20% sucrose overnight at 4 °C. The following day, muscles were embedded in OCT medium (NEG-50; Richard Allen Scientific) and froze in liquid N_2_-chilled 2-methylbutane. 10 μm cryosections were placed on Superfrost plus slides, and sections were stained with hematoxylin and eosin. Images of tissue sections were taken on a Nikon Eclipse Ni-U widefield epifluorescence microscope equipped with a Nikon DS-F12 camera. For the fiber analyses, images were enumerated and quantitated using ImageJ.

### Pax7 immunofluorescence of tissue sections

Non-injured tibialis anterior muscles were dissected, processed, and cryosectioned, as described above. Sections were permeabilized in 0.5% Triton X-100/PBS, washed with PBS, and underwent heat-mediated antigen retrieval with EDTA buffer (1 mM EDTA, pH 8.0, 0.05% Tween-20). Following avidin/biotin blocking (Vector Labs), mouse IgGs were blocked with mouse on mouse IgG blocking reagent (Vector Labs). Sections were then further blocked with 3%BSA/0.1% Triton X-100/PBS before addition of primary antibodies [Pax7 (PAX7) mouse monoclonal; Santa Cruz; 1/20; GFP-tag rabbit polyclonal; ThermoFisher; 1/250] in 3%BSA/0.1% Triton X-100/PBS overnight at 4 **°**C. The following day, sections were washed and stained with Alexa Fluor 488 goat anti-Rabbit IgG (1/250; for EGFP), Alexa Fluor 555-conjugated wheat germ agglutinin (WGA; ThermoFisher; 1 μg/mL; to stain the extracellular matrix), and Alexa Fluor 647-conjugated streptavidin (BioLegend; 1/250; to mark Pax7^+^ cells) in 3% BSA/0.1% Triton X-100/PBS at room temperature. After washing with PBS, sections were stained with 20 μg/mL DAPI and coverslips were mounted with Prolong diamond antifade reagent (ThermoFisher). For imaging for the presence of GFP in tissue sections without antibody staining, muscle was processed as above up until after sectioning and stained with 20 μg/mL DAPI. Coverslips were mounted with Prolong diamond antifade reagent (ThermoFisher).

### Whole muscle imaging

The tibialis anterior muscle was mounted on a custom made platform, and a coverslip was lowered from the top to make contact with the exposed muscle. High-resolution serial optical sections were acquired with an Olympus FV1200MPE microscope equipped with a Chameleon Vision II 2-photon laser. A laser beam that was tuned at 890 nm was focused through a × 10 objective lens (Olympus UPLSAPO, N.A. 0.4) and scanned with a field of view of 1270 μm^2^. The emission light was then spectrally separated into green (495–540 nm) and red (575-630 nm) channels and collected by two GaAsP photo-detectors. The following acquisition settings were used for the green and red photo-detectors, respectively: HV: 550/525 V; Gain 1/1, Offset: 3/4%. Serial optical sections were acquired in 4 μm steps for total range of 200 μm from the surface of the muscle. Muscle fibers were marked by the appearance of Second Harmonic Generation (SHG) in red channel [37]. Muscle stem cells marked with EGFP were clearly visible in the green channel.

### Statistical analysis

Significance was determined using two-tailed unpaired *t* tests with Welch’s correction using GraphPad Prism 6 software. *p < 0.05; **p < 0.01; ***p < 0.001; NS = not significant. Other procedures not listed here can be found in the Supplementary methods section (Additional file 4).

## Results

### Generation of a Pax7EGFP mouse and transgene expression

**Fig. 1:**
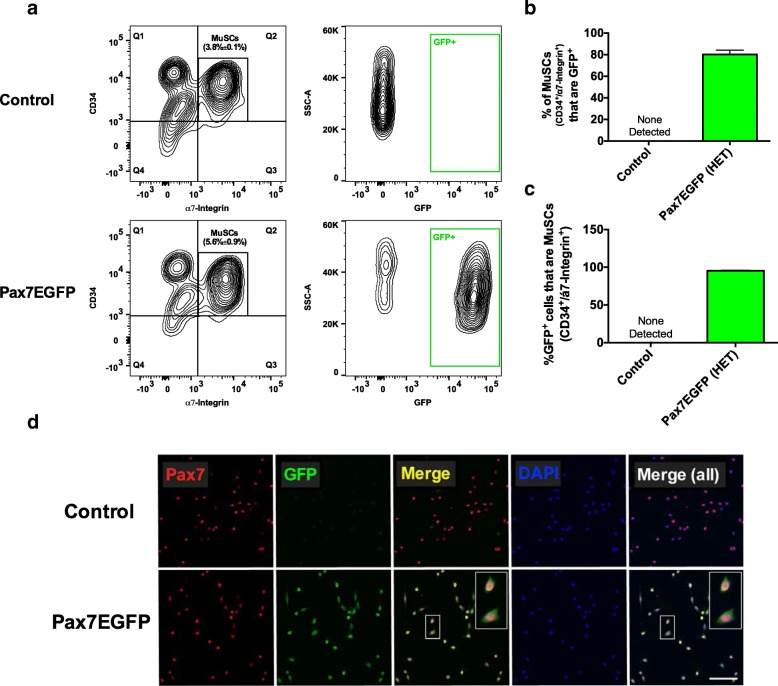
EGFP expression in MuSCs of Pax7EGFP muscles. **a** Representative flow cytometry plots of MuSC populations (CD11b^−^/CD31^−^/CD45^−^/Sca1^−^/CD34^+^/α7-integrin^+^) derived from control (Pax7EGFP negative) and Pax7EGFP mice (left). MuSCs were then analyzed for the presence of GFP (right). **b** Assessment of the percent of MuSCs from (**a**) that are GFP^+^. **c** Analysis of GFP^+^ cells that are also CD34^+^ and α7-integrin^+^. **d** Immunofluorescence staining of FACS-isolated MuSCs for Pax7 and GFP show all cells co-express both markers. Scale bar = 100 μm. *n* > 3 mice per genotype

The Pax7EGFP mouse was generated by cloning of the EGFP gene into the first exon of the Pax7 gene into a BAC genomic clone (RP23-204F20, obtained from ImaGenes; Additional file 1: Figure S1a), using ET recombination [38]. The resulting construct (Pax7-EGFP) uses the endogenous Pax7 promoter driving expression of EGFP and utilizes its endogenous putative proximal and distal enhancer elements. The Pax7-EGFP targeting vector was microinjected into fertilized eggs of C57Bl/6 female mice. More than 150 offspring were genotyped for EGFP integration, which ultimately yielded five possible transgenic mouse lines based on the presence of the GFP transgene (Additional file 1: Figure S1b). Since Pax7 expression is robust in quiescent MuSCs [17], we next sought to determine whether the lines deemed positive in our PCR screen correlated with expression of the transgene in MuSCs. For this purpose, the five positive lines (#1, #3, #4, #5, #6) were expanded. MuSCs isolated from adult skeletal muscles were further screened for EGFP expression by flow cytometry (Table 1). Transgenic mouse line #5, which displayed the most robust EGFP signal by FACS analysis (Table 1 and Additional file 5: Figure S4) was the founder used to generate the Pax7EGFP colony. To determine the exact number of the transgene integrations into the genome of this line, we used a copy number variation assay. Our analysis showed integration of the transgene into two sites in a heterozygous mouse, and four sites in a homozygous mouse (Additional file 2: Figure S2a). The number of integrations correlates well with the high integrity and expression of EGFP observed in muscle stem cells of the mouse line (Fig. 1a). However, we have found a reduction in MuSC number in Pax7EGFP homozygotes (Additional file 2: Figure S2b), presumably due to the increased number of integrations that often increases instability [39]. Thus, analyses of MuSCs were conducted on heterozygous Pax7EGFP mice.

**Table 1:** Screening of transgenic mouse lines

Mouse line	EGFP expression (FACS analysis)
Pax7EGFP-1	Negative
Pax7EGFP-3	Very low
Pax7EGFP-4	Negative
Pax7EGFP-5	HIGH
Pax7EGFP-6	Negative

### Isolation of GFP^+^ cells from Pax7EGFP muscles reveals labeling of MuSCs with high fidelity

To test the strength of the fluorescence signal in our Pax7EGFP MuSCs, we further characterized them by FACS analysis and found robust expression (> 3 orders of magnitude) of EGFP in the MuSC population (defined as lineage^−^CD34^+^/α7-integrin^+^), while control (Pax7EGFP negative) MuSCs expressed no GFP (Fig. 1a and Additional file 5: Figure S4). To determine whether the specificity of EGFP is confined to MuSCs, the proportion of isolated MuSCs that expressed EGFP was determined by flow cytometry. Our analysis demonstrates that > 80% of MuSCs (CD34^+^/α7-integrin^+^) derived from Pax7EGFP mice (1.5–3 months old) express EGFP (Fig. 1b and Additional file 5: Figure S4). In addition, no EGFP expression was observed in the CD34^−^/ α7-integrin^−^/Sca1^+^/CD11b^+^/CD31^+^/CD45^+^ populations in these mice (Additional file 5: Figure S4). As an additional control, we included Pax7Cre/Rosa^mTmG^ mice, where GFP expression depends on Pax7-driven Cre recombination. Similar to what we observed with our Pax7EGFP mouse line, ~ 80% of CD34^+^/α7-integrin^+^ cells expressed GFP (Additional file 3: Figure S3). This consistency demonstrates the specificity of our Pax7EGFP mouse in labeling MuSCs. Further characterization of the Pax7EGFP MuSCs shows that all of the EGFP^+^ cells (~ 100%) derived from the Pax7EGFP mouse were also MuSCs (CD34^+^/α7-integrin^+^) (Fig. 1c), demonstrating that the EGFP^+^ cells derived from the Pax7EGFP line are an ample source of MuSCs that are also CD34^+^/α7-integrin^+^. We next assayed the number of EGFP^+^ cells that are also Pax7^+^ in vitro and found that the EGFP^+^ cells also co-stained for Pax7 (Fig. 1d), indicating a high specificity of EGFP in labeling MuSCs. Altogether, these data suggest that the Pax7EGFP mouse is a tool for straightforward isolation of MuSCs, using the EGFP signal.

### Pax7EGFP- and wild-type-derived MuSCs have similar myogenic properties

**Fig. 2:**
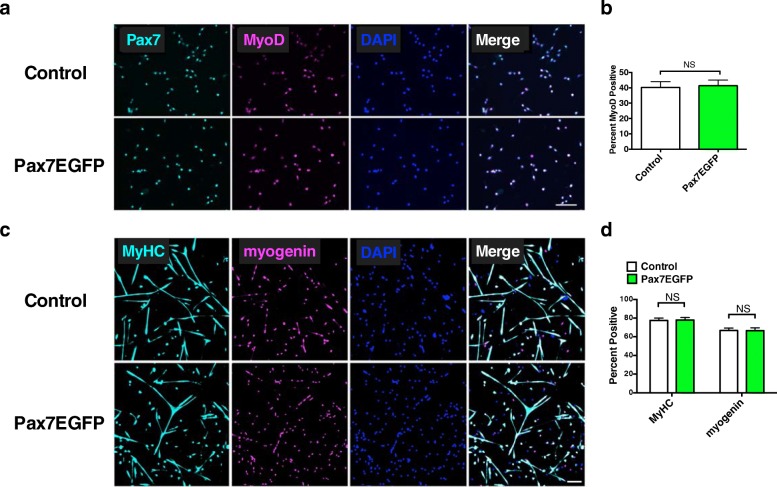
Myogenic potential of Pax7EGFP MuSCs in vitro. **a** MuSCs from either genotype were plated on laminin-coated chamber slides in growth medium for 2 days, prior to fixation and staining for Pax7 and MyoD. Scale bar = 100 μm. **b** Quantitation of MyoD positively stained cells in (**a**). *n* ≥ 3 mice per genotype and *N* > 500 cells per condition analyzed. **c** MuSCs from control or Pax7EGFP mice were grown first in proliferation medium for 5 days and then switched to differentiation medium for 2 days, fixed, and stained for the differentiation markers Myosin Heavy Chain (MyHC) and myogenin. Scale bar = 100 μm. **d** Quantitation of data displayed above. *n* ≥ 3 mice analyzed per group with *N* > 1000 cells analyzed per group

To further explore the myogenic performance of Pax7EGFP cells, we evaluated the expression levels of the early myogenic marker, MyoD, and found no difference between groups (Fig. 2a, b), suggesting similar myogenic capabilities between Pax7EGFP and wild-type MuSCs. EdU incorporation analysis and TUNEL staining also confirmed the similarity in proliferation capacity and cell death of these cells in vitro (Additional file 6: Figure S5a–d). These results are consistent with a previous report that showed constitutive expression of Pax7 in satellite cell-derived myoblasts did not affect MyoD expression or proliferation [17]. To assess the effect of the Pax7EGFP transgene on MuSC differentiation, the expression of two established later myogenic markers, myogenin, and myosin heavy chain (MyHC), were queried (Fig. 2c). The percentage of positive cells for these markers were not significantly different between cultures derived from Pax7EGFP-mice compared with wild-type cultures (Fig. 2d), indicating that there is no differentiation impairment of Pax7EGFP cells. Together, the identical proliferation and differentiation properties between Pax7EGFP and wild-type MuSCs confirms that the transgene does not interfere with the typical myogenesis process in vitro.

### Visualization by fluorescence microscopy reveals that Pax7-driven EGFP is a dynamic marker of MuSC stemness

**Fig. 3:**
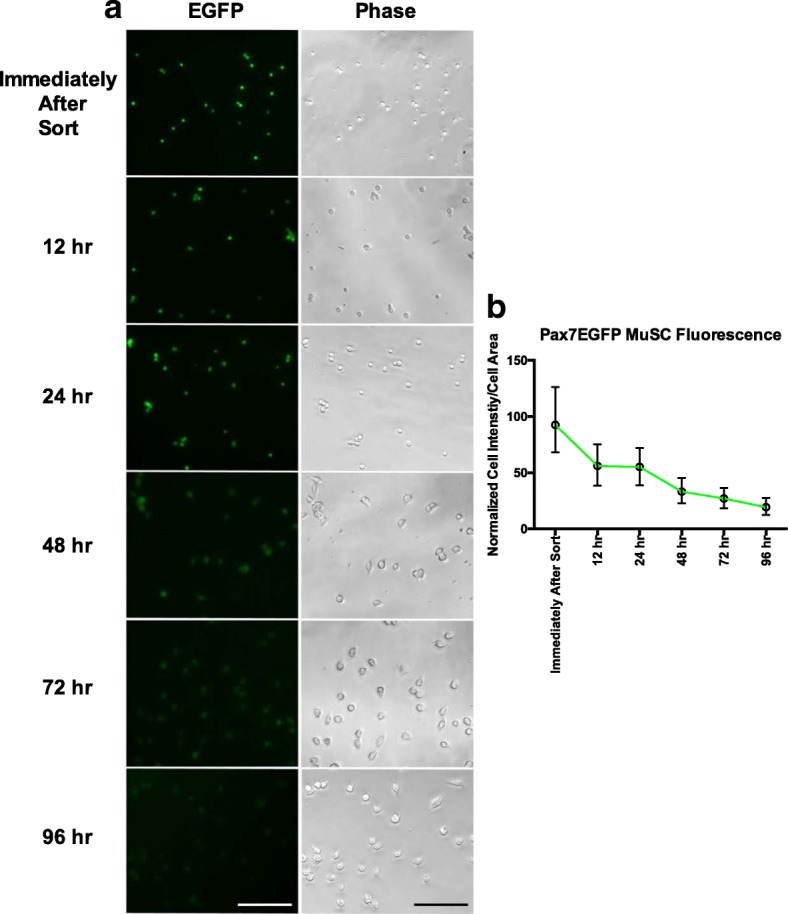
GFP expression in Pax7EGFP-derived MuSCs is dynamic. **a** FACS-sorted Pax7EGFP MuSCs were imaged daily for the presence of EGFP for 4 days. Scale bar = 100 μm. **b** Quantification of the signal intensities of Pax7EGFP MuSCs, with cell size taken into account, and normalized to the percent fluorescence intensity observed after initial plating. MuSCs were individually analyzed from *n* = 3 Pax7EGFP heterozygous mice. Displayed as median with interquartile range

The myogenic program is orchestrated by the progression from quiescence, activation, proliferation, and differentiation (reviewed in [18, 40]). Quiescent MuSCs express the transcription factor Pax7 [2]. Upon activation, MuSCs proliferate, downregulate Pax7, and differentiate [17, 18]. To verify the precision of our Pax7-driven EGFP mice in mirroring what occurs during a typical myogenesis process, we monitored the levels of EGFP expression over time (Fig. 3a). Interestingly, we found that the expression of EGFP decreases shortly after plating (Fig. 3b), as expected in cells that are in transition from a quiescent state towards a more activated/committed phase [17, 18]. The temporal expression pattern of Pax7EGFP-derived MuSCs recapitulates the reported Pax7 expression pattern of MuSCs [2, 17, 18, 40–43] and demonstrates the feasibility of using the EGFP signal in our transgenic mouse as a dynamic marker of MuSCs.

### Robust and persistent Pax7EGFP expression in embryos and adult tissues

**Fig. 4:**
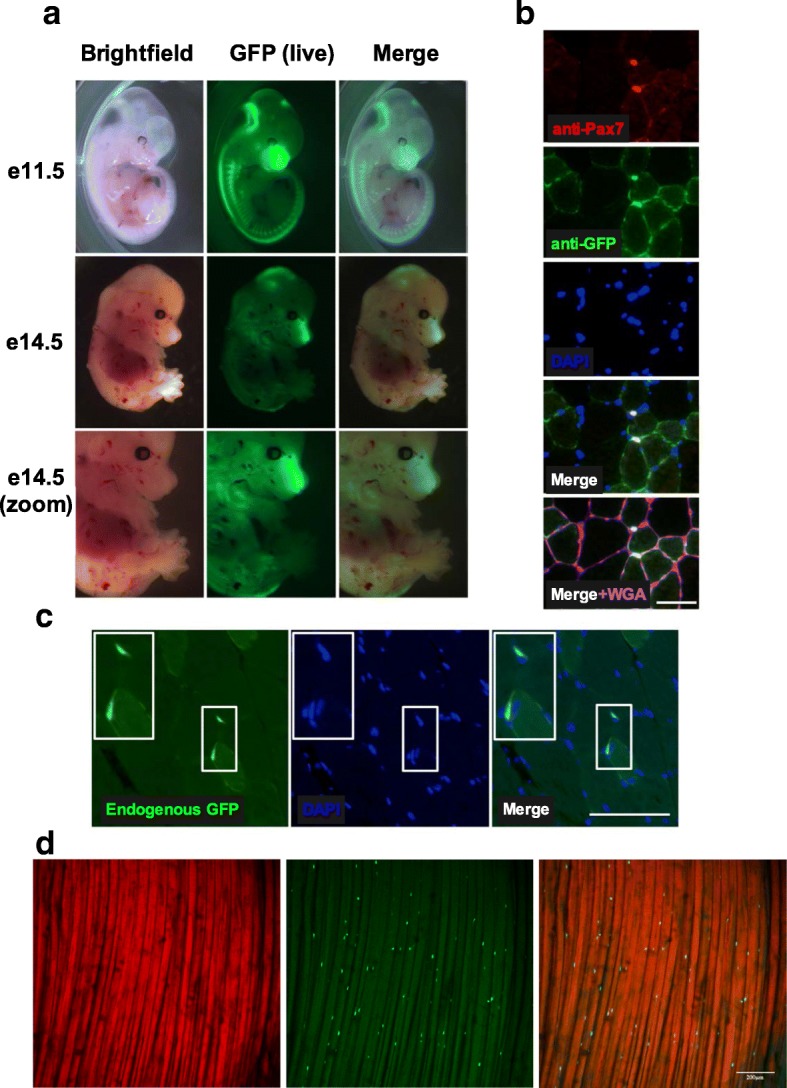
GFP expression patterns in tissues of Pax7EGFP mice. **a** Whole embryos were isolated from timed matings of Pax7EGFP heterozygous mice with wild-type mice and imaged for green fluorescence. Images were merged with brightfield images. Top: e11.5 dpc embryos. Note expression of GFP in the nasal region and somites. Middle: imaging of e14.5 dpc embryos. Bottom: close-up image of e14.5 dpc embryos demonstrating expression in the forelimb and hindlimb musculature. **b** Pax7 and GFP co-localize in vivo. Non-injured TA muscles were fixed and stained with antibodies against Pax7 and GFP, and co-stained with both DAPI and fluorescently tagged-WGA (ECM). Scale bar = 50 μm. Note the clear overlap in expression. Data were confirmed in *n* > 4 mice. **c** EGFP signal is present in TA tissue sections of Pax7EGFP mice without the need to stain with GFP antibody. **d** Two photon imaging of the tibialis anterior (longitudinal) of live Pax7EGFP mice in vivo. Left: muscle fibers. Middle: Pax7EGFP positive cells. Right: Merged image. Note the distinct GFP signal present in the satellite cell position. Scale bar = 200 μm

To test whether the EGFP expression pattern in Pax7EGFP mice follows that of the endogenous Pax7 protein during development, we analyzed whole embryos at two representative embryonic days (e11.5 and e14.5). EGFP expression was clearly observed in the somites, frontonasal region, and neural tube in e11.5 dpc embryos, while expression was found in the brain, frontonasal region, and skeletal muscles of the arms and legs in e14.5 dpc embryos (Fig. 4a). This analysis demonstrates that the EGFP expression pattern in the Pax7EGFP mice replicates the spatiotemporal expression that was previously reported for Pax7 [10, 12, 43–45] and confirms that Pax7EGFP is a functional equivalent reporter of Pax7.

To determine the specificity of Pax7 promoter-driven GFP expression in adult skeletal muscles, the localization of Pax7 and GFP was determined in cryosections by immunofluorescence microscopy. We consistently observed colocalization of Pax7 staining with GFP staining in adult Pax7EGFP muscles (Fig. 4b, Additional file 7: Figure S6). Importantly, in our mouse line the EGFP is evident under fluorescence microscopy post-fixation, even without the use of a GFP antibody (Fig. 4c). The usefulness of our Pax7EGFP mouse is further demonstrated by in vivo imaging of MuSCs in whole muscles. Specifically, by using two-photon microscopy, we found that EGFP is detectable in the satellite cell position within the skeletal muscles of Pax7EGFP mice (Fig. 4d). In summary, these data demonstrate the specificity of EGFP expression driven by the Pax7 promoter in our mouse line and highlights its bright expression in the satellite cell position of live and fixed adult skeletal muscles.

### Typical skeletal muscle homeostasis and regeneration abilities in Pax7EGFP mice

**Fig. 5:**
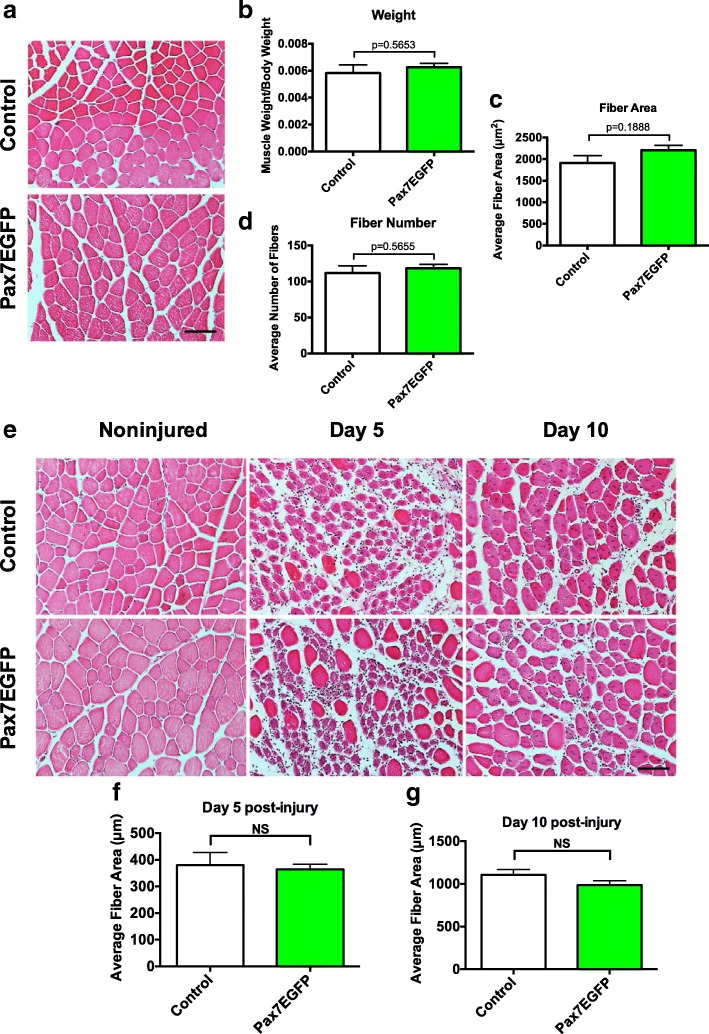
Histological analysis of Pax7EGFP mice. **a** Representative images of gastrocnemius muscles from control (Pax7EGFP negative, top) or Pax7EGFP heterozygous mice (Bottom) stained with hematoxylin and eosin. **b** Quantitation of the gastrocnemius muscle weight normalized to body weight of each mouse. Further analyses included analysis of (**c**) fiber area of the gastrocnemius muscles between genotypes and (**d**) number of muscle fibers per section. *n* ≥ 3 age and gender-matched mice per genotype per condition. **e** Representative images of control and Pax7EGFP tibialis anterior muscles following injury. Tibialis anterior muscles were injured with notexin or left non-injured (left), and muscles were harvested 5 (middle) or 10 (right) days post-injury. Muscles were cryosectioned and stained with hematoxylin and eosin and imaged. Scale bar = 100 μm. **f** and **g** Quantitation of centrally nucleated fibers. *n* ≥ 3 mice per genotype per condition. *N* > 500 fibers analyzed per genotype per condition

We previously showed that the insertion of the Pax7-EGFP transgene in the genome does not affect the myogenic progress in vitro (Fig. 2). To further evaluate if such a possibility exists in vivo, the gastrocnemius, quadriceps, and tibialis anterior muscles of Pax7EGFP heterozygous or control (Pax7EGFP negative) mice were harvested and weighed, prior to histological staining. Muscle weights were normalized to total body weight of the harvested mouse to account for any potential abnormalities resulting from mouse size/age. We found no overt differences in the morphologies (Fig. 5a), muscle weight (Fig. 5b), fiber area (Fig. 5c), or number of fibers (Fig. 5d) in all examined skeletal muscles between control and Pax7EGFP^+^ mice (Additional file 8: Figure S7). To evaluate the behavior of Pax7EGFP mice under regeneration conditions, we induced muscle injury with notexin. Histological examination of cross-sections (Fig. 5e) revealed no apparent defects in regeneration either 5 or 10 days after injury (Fig. 5f, g). In aggregate, these data demonstrate that Pax7EGFP mice appear morphologically similar to controls, both in homeostatic and injury conditions, which argues against a positional effect arising from the transgene insertion. Finally, to confirm that Pax7EGFP-labeled cells function as MuSCs during regeneration, we stained tibialis anterior cryosections from non-injured or notexin-injured Pax7EGFP heterozygous mice with Pax7 and GFP antibodies and analyzed the level of overlap between Pax7^+^ cells and GFP^+^ cells (Additional file 9: Figure S8a). We found substantial overlap between these cell populations (Additional file 9: Figure S8b) in the early stages (D5) and later stages (D10) of regeneration. These data reveal that GFP^+^ cells from the Pax7EGFP mouse label MuSCs and function in a typical manner during the regeneration process.

## Discussion

Pax7 is the most utilized marker of quiescent satellite cells [18]. Pax7 labels all stem cells in the adult muscle with myogenic potential [46]. Although previously generated Pax7 reporter lines [19, 44] have yielded important insights of muscle stem cells using prospective cell isolation methods, they failed to mark Pax7^+^ cells in vivo (native and live) in adult mice. Here, we showed the successful generation and detailed characterization of a new transgenic mouse with a bright EGFP fluorescence, which is optimal for labeling Pax7^+^ cells in vivo. The overall stronger in vivo expression of our Pax7EGFP line compared to the Pax7zsGreen line could be explained by the differences between a monomeric GFP and a cytoplasmic tetrameric protein [47, 48], the different BAC clones used to generate the targeting vector and/or differences of the integration site. Similarly, the different BAC clone used to generate the Pax7-nGFP line [19, 49] is likely to impact the levels of EGFP live expression. A common obstacle in generating transgenic mice from BAC chromosomes has been the poor expression of the transgene due to positional effects [50]. The high expression of EGFP in the Pax7EGFP line argues against integration into a heterochromatin region of the genome. Due to the technical inability to locate the defined genomic locus of the BAC insertions [50], we determined the number of insertions and further analyzed the myogenic properties of Pax7EGFP muscles. The normal in vitro myogenic potential together with the ordinary muscle morphology and in vivo regeneration potential exclude the possibility that the BAC integration is affecting a muscle-relevant gene.

To fully characterize the properties of Pax7EGFP cells, we have isolated MuSCs using α7-integrin and CD34 cell surface markers and we have identified that > 80% of MuSCs were also positive for GFP expression. These data are in line with research that displayed similar percentages, when Pax7-positive cells were assayed for different typical cell surface markers used in the isolation of MuSCs, including CXCR4/β1-integrin, VCAM, and α7-integrin/CD34 [22]. Importantly, we found that all EGFP^+^ cells in the Pax7EGFP muscles are also Pax7^+^, as expected if all necessary sequences in the promoter region that required to establish the native Pax7 genetic organization are present. While flow cytometry is a very sensitive tool, fluorescence signals detectable by FACS are not always strong enough to be identified by microscopy at the same time [51], limiting the usefulness of a reporter line. To assess the intensity of EGFP in our Pax7EGFP mice, we plated and imaged isolated MuSCs by fluorescence microscopy. We found that the EGFP expression was very bright in all isolated cells, demonstrating that the levels of GFP expression are high and detectable by both flow cytometry and fluorescence microscopy.

Despite a recent debate regarding the exact role of Pax7 in adult muscles [32–34], the ability of Pax7 expressing stem cells to contribute to skeletal muscle regeneration is indisputable. However, the Pax7 mechanisms that preserve stem cell homeostasis in uninjured tissues, its role as a chromatin modifier and its requirements in resident non-muscle cells remain to be elucidated (discussed in [35]). Our analysis revealed that the EGFP in the Pax7EGFP line is dynamic, recapitulating the temporal endogenous Pax7 expression pattern. Although the Pax7EGFP mouse cannot be used to follow cells that then lose their stemness and downregulate Pax7 (therefore become EGFP negative), it could be extremely useful to follow the molecular physiology of Pax7 in in vivo self-renewal, heterogeneity, and asymmetric cell division of MuSCs. In addition, a recent study reported an unexpected role of Pax7 in maintenance of the heterochromatin state [32]. It would be interesting to analyze in detail these properties using the Pax7EGFP line in conjunction with histone markers. Furthermore, it has been reported that muscle resident non-satellite cells, such as PW1^+^ cells [52, 53] or pericytes [54], transiently activate the Pax7 promoter and participate in muscle repair. Our Pax7EGFP line in combination with specific markers for these populations will be useful in following these transient populations as they contribute to muscle repair. Finally, MuSC dysregulation is a physiologically relevant topic, particularly in degenerative muscle diseases, where stem cell defects lead the disease progression [55–57].

Overall, we show that our mouse line drives EGFP expression in a cell-specific manner that is easily detectable and has informative physiological effects. We propose that this mouse model will be beneficial for anyone studying muscle stem cell motility and/or regeneration in an in vivo setting. Given the intense fluorescent signal, this mouse could be used to visualize MuSCs in a steady state to understand their cell-autonomous function, as well as how they migrate and behave following injury.

## Conclusion

Here, we have described the creation and characterization of a new mouse model, where EGFP fluorescence is driven by Pax7 expression. We found EGFP expression to be robust and dynamic, and the EGFP labeled cells can be easily isolated by flow cytometry and/or followed using live imaging in vivo. We conclude that EGFP is a reliable tool for convenient monitoring of Pax7^+^ cells, and we propose that this mouse line can be of great benefit to the satellite cell/MuSC field to understand the role of Pax7 in stem cell homeostasis, heterogeneity, and asymmetric cell division in healthy and/or disease conditions.

## Additional files

Additional file 1: **Figure S1.** Generation of the Pax7EGFP mouse. (a) Schematic of the Pax7-EGFP targeting BAC that was used to generate the Pax7EGFP line. (b) Agarose gel depicting PCR-genotyped mouse lines from Table 1. A mouse carrying the Pax7EGFP transgene yields a single product of 706 bp. Control (wild-type) mice produce no band at this size. (PDF 442 kb)

Additional file 2: **Figure S2.** Pax7EGFP copy number variation analysis and in-depth genotyping, and effect of transgene integrations on MuSCs. (a) Genomic DNA was isolated and purified from three Rosa^mtmg^ /Pax7Cre heterozygous mice (containing 1 GFP genomic copy), as well as from two mice each of wild-type, Pax7EGFP heterozygous, and Pax7EGFP homozygous backgrounds (one from each gender). Purified DNA was subjected to a TaqMan Copy Number Variation Assay, according to the manufacturer’s instructions. Data were normalized to the one copy number present in the Rosa^mT/mG^ /Pax7 Cre heterozygous mice. (b) MuSCs were isolated from Pax7EGFP heterozygous or homozygous mice by FACS. Note the reduction of MuSC numbers in homozygotes. (PDF 123 kb)

Additional file 3: **Figure S3.** MuSCs from Pax7EGFP mice are similar to Pax7-labeled MuSCs. (a) MuSCs were isolated as in Fig. 1 from Pax7EGFP heterozygous mice and Rosa^mTmG^/Pax7Cre dual heterozygous mice. (b) Evaluation of the percent of MuSCs in (a) that are also EGFP^+^. (PDF 316 kb)

Additional file 4: Supplementary methods [56, 58]. (DOCX 23 kb)

Additional file 5: **Figure S4.** FACS schematic of MuSC isolation. Top: gating strategy for the gate selection of parent populations of muscle cell isolates, singlets, and live cells (7-AAD negative). Measurement of GFP^+^ cells in lineage positive cell populations (Sca1^+^/CD11b^+^/CD31^+^/CD45^+^) showed no GFP expression (red). Bottom: MuSC enrichment by gating CD11b^−^/CD45^−^/CD31^−^/Sca1^−^ (lineage negative) populations followed by gating for CD34^+^/α7-integrin^+^ and finally the populations of GFP^+^ cells from Pax7EGFP mice (green) or control mice (cyan) was displayed as histograms. (PDF 3145 kb)

Additional file 6: **Figure S5.** Analysis of MuSC proliferation and cell death. (a) Measurement of proliferative capacity in MuSCs derived from control or Pax7EGFP mice. FACS-sorted MuSCs were plated on laminin-coated chamber slides in myoblast media containing bFGF for 2 days. EdU was added to the culture media, and cells were incubated for 2 h. Cells were fixed, and EdU incorporation was assayed by fluorescence microscopy. As a control, some cells were not treated with EdU. Scale bar = 100 μm. (b) Quantitation of data shown in (a). *n* ≥ 3 mice assayed, with at least *N* = 700 cells analyzed per group. (c) Measurement of cell death levels in FACS-sorted MuSCs by TUNEL assay. Cells were plated as in (a) and processed as described in the methods. As a positive control, fixed cells were treated with DNase. Scale bar = 100 μm (d) Quantitation of cell death levels shown in (c). ND, none detected. *n* ≥ 3 mice were analyzed; *N* > 400 cells were analyzed. (PDF 3145 kb)

Additional file 7: **Figure S6.** Technical immunofluorescence control for Fig. 4b. (Left) Pax7EGFP negative mouse TA muscle cryosections were stained with Pax7 and GFP antibodies. (Right) TA muscle cryosections of Pax7EGFP mice processed the same as control muscle tissue, with the exception of no Pax7 or GFP antibody staining occurred. Note: while the GFP signal of Pax7EGFP mice survives our fixation protocol for cryosectioning, it does not survive EDTA-mediated antigen retrieval necessary for Pax7 staining. (PDF 89 kb)

Additional file 8: **Figure S7.** Histological analysis of additional Pax7EGFP muscles. Representative images of quadriceps (a) and tibialis anterior (b) muscles from non-injured control (top) or Pax7EGFP heterozygous mice (bottom) stained with hematoxylin and eosin. (c–d) Individual muscle weights were normalized to total body weight per mouse. (e–f) Analysis of muscle fiber number. (g–h) Quantification of muscle fiber area. *n* = at least 3 mice (age and gender-matched) per genotype per condition. (PDF 180 kb)

Additional file 9: **Figure S8.** Overlap of Pax7^+^ and GFP^+^ cells during regeneration. (a) Imaging of tibialis anterior cryosections stained with Pax7 and GFP under conditions of no injury, or 5 and 10 days post-injury. (b) Quantification of Pax7^+^ cells that overlap with GFP^+^ cells. *n* = 2–3 mice per condition. Displayed is the mean ± standard deviation. (PDF 605 kb)

## Abbreviations

AAD: 7-aminoactinomycin D
bFGF: Basic fibroblast growth factor
DAPI: 4′,6-diamidino-2-phenylindole
DMEM: Dulbecco’s modified Eagle’s medium
DNA: Deoxyribonucleic acid
ECM: Extracellular matrix
EDTA: Ethylenediaminetetraacetic Acid
EdU: 5-Ethynyl-2′-deoxyuridine
EGFP: Enhanced green fluorescent protein
FBS: Fetal bovine serum
MuSCs: Muscle stem cells
MyHC: Myosin heavy chain 3
OCT: Optimal cutting temperature
Pax7: Paired box 7
PBS: Phosphate-buffered saline
PFA: Paraformaldehyde
TA: Tibialis anterior
Tfrc: Transferrin receptor
TUNEL: Terminal deoxynucleotidyl transferase dUTP nick end labeling
WGA: Wheat germ agglutinin
