# Supplementary figures and images for: A robust Pax7EGFP mouse that enables the visualization of dynamic behaviors of muscle stem cells
Source: Skelet Muscle. 2018 Aug 24;8:27. doi: 10.1186/s13395-018-0169-7 (PMC6107960; doi:10.1186/s13395-018-0169-7)

Figure S1

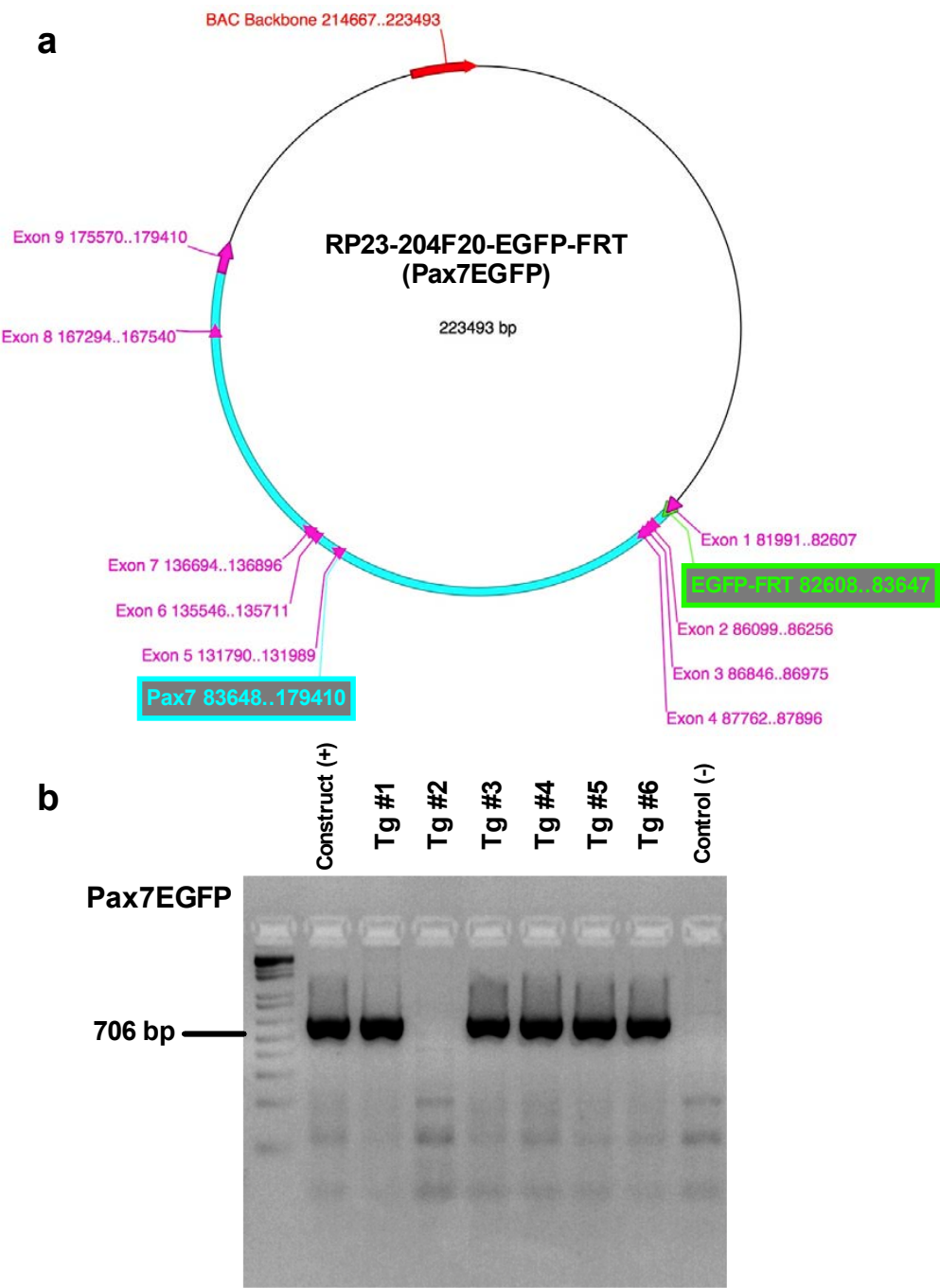

Supplement: Supplementary file 1 — Figure S1. Generation of the Pax7EGFP mouse. (a) Schematic of the Pax7-EGFP targeting BAC that was used to generate the Pax7EGFP line. (b) Agarose gel depicting PCR-genotyped mouse lines from Table 1. A mouse carrying the Pax7EGFP transgene yields a single product of 706 bp. Control (wild-type) mice produce no band at this size. (PDF 442 kb) [file 13395_2018_169_MOESM1_ESM.pdf]

Figure S2

a

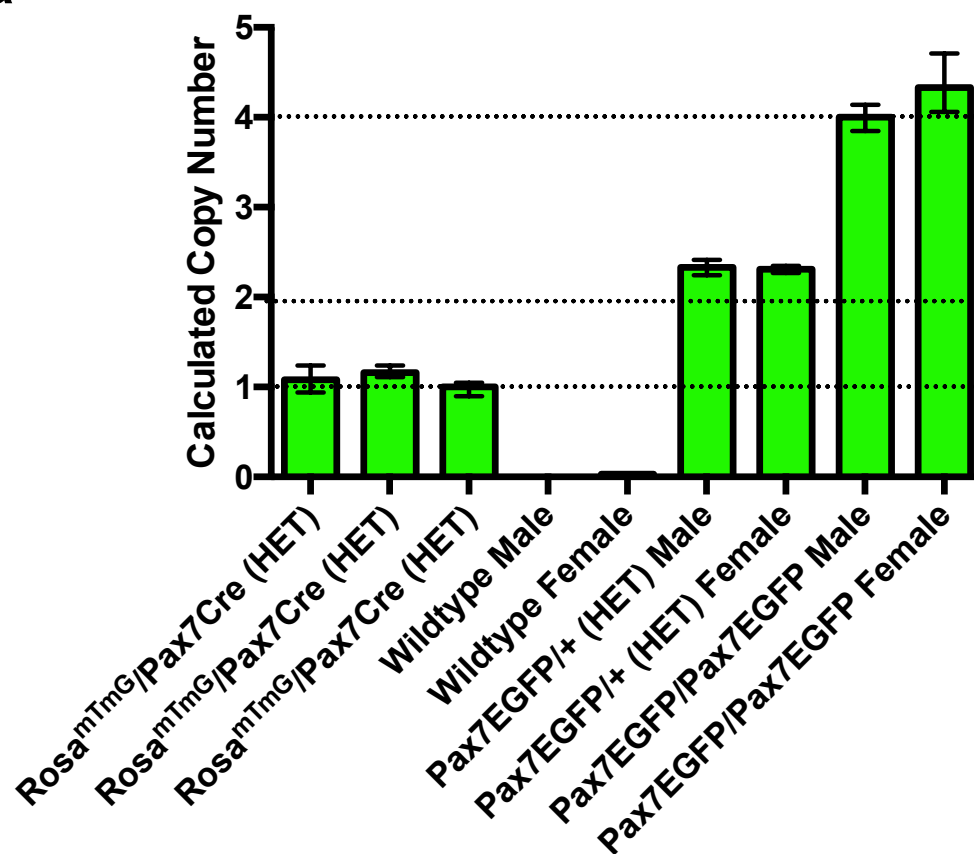

b

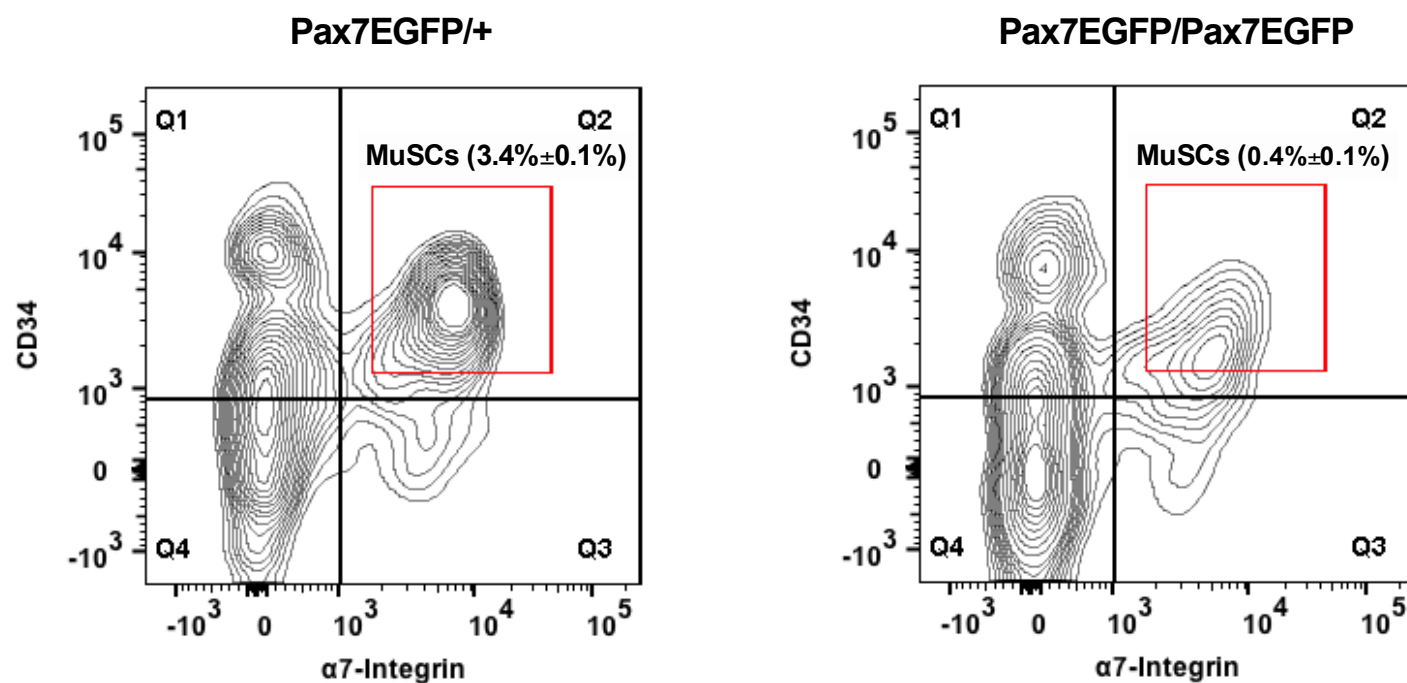

Supplement: Supplementary file 2 — Figure S2. Pax7EGFP copy number variation analysis and in-depth genotyping, and effect of transgene integrations on MuSCs. (a) Genomic DNA was isolated and purified from three Rosamtmg /Pax7Cre heterozygous mice (containing 1 GFP genomic copy), as well as from two mice each of wild-type, Pax7EGFP heterozygous, and Pax7EGFP homozygous backgrounds (one from each gender). Purified DNA was subjected to a TaqMan Copy Number Variation Assay, according to the manufacturer’s instructions. Data were normalized to the one copy number present in the RosamT/mG /Pax7 Cre heterozygous mice. (b) MuSCs were isolated from Pax7EGFP heterozygous or homozygous mice by FACS. Note the reduction of MuSC numbers in homozygotes. (PDF 123 kb) [file 13395_2018_169_MOESM2_ESM.pdf]

Figure S3

a

Pax7EGFP

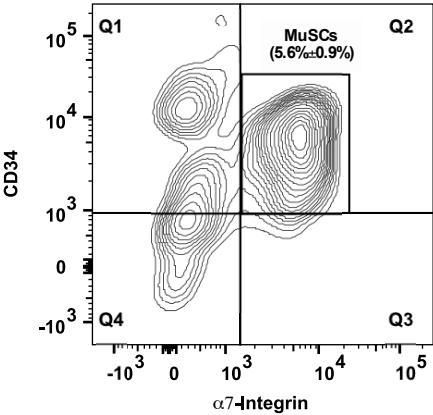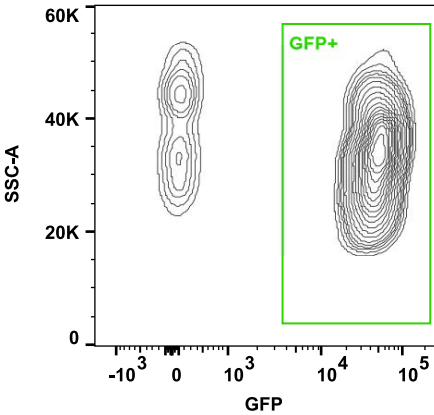

Rosa<sup>mTmG</sup>/  
Pax7Cre

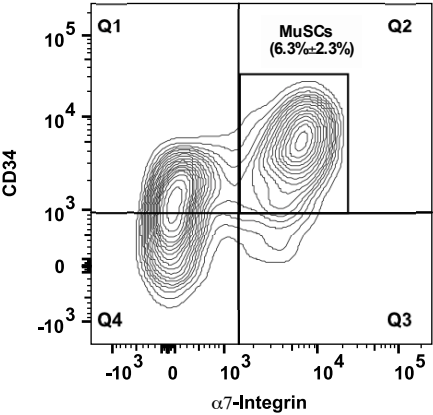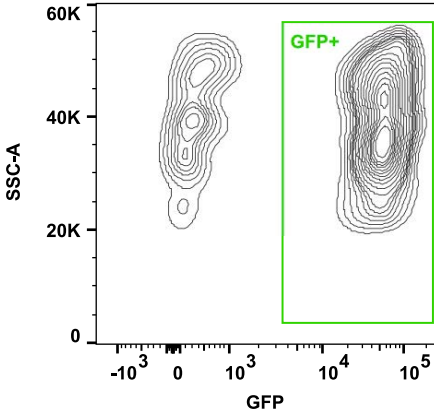

b

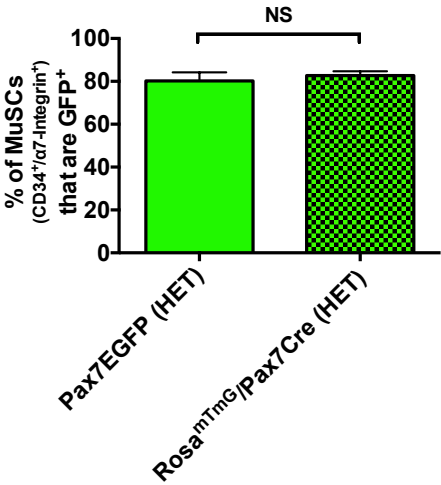

Supplement: Supplementary file 3 — Figure S3. MuSCs from Pax7EGFP mice are similar to Pax7-labeled MuSCs. (a) MuSCs were isolated as in Fig. 1 from Pax7EGFP heterozygous mice and RosamTmG/Pax7Cre dual heterozygous mice. (b) Evaluation of the percent of MuSCs in (a) that are also EGFP+. (PDF 316 kb) [file 13395_2018_169_MOESM3_ESM.pdf]

Figure S4

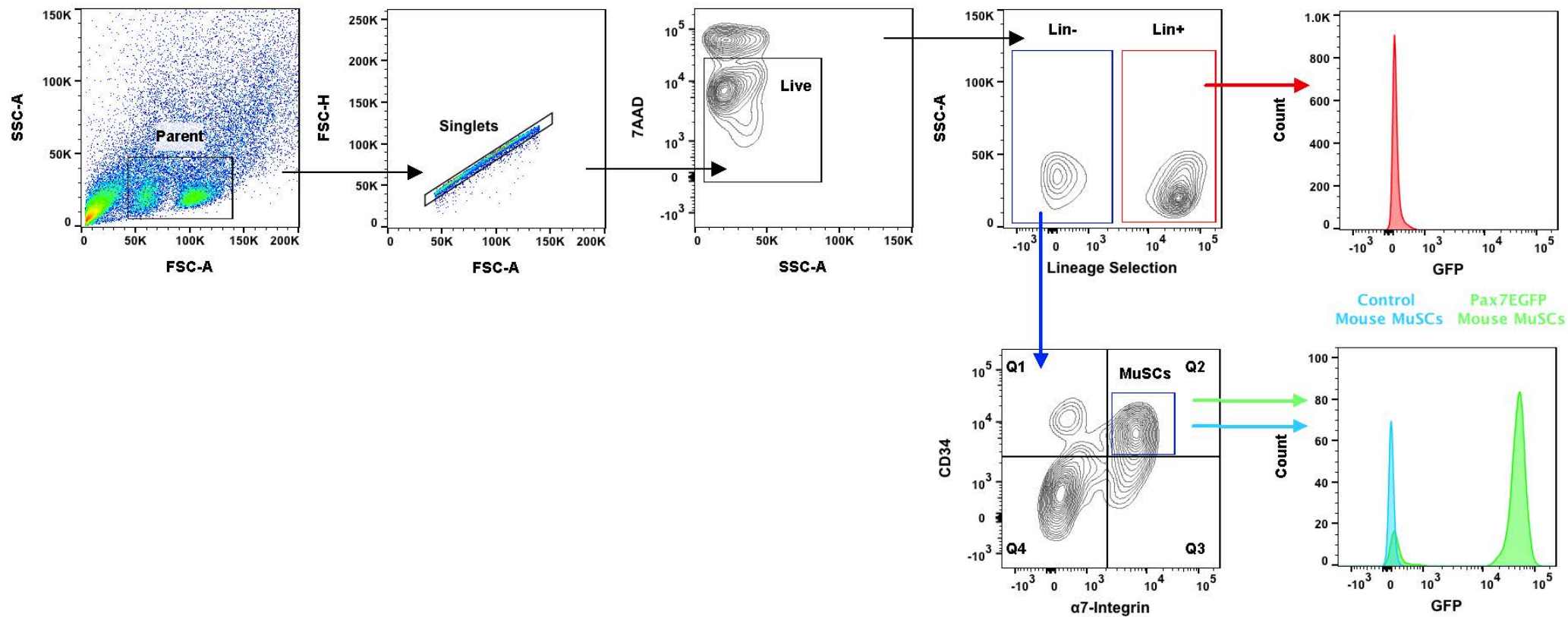

Supplement: Supplementary file 5 — Figure S4. FACS schematic of MuSC isolation. Top: gating strategy for the gate selection of parent populations of muscle cell isolates, singlets, and live cells (7-AAD negative). Measurement of GFP+ cells in lineage positive cell populations (Sca1+/CD11b+/CD31+/CD45+) showed no GFP expression (red). Bottom: MuSC enrichment by gating CD11b−/CD45−/CD31−/Sca1− (lineage negative) populations followed by gating for CD34+/α7-integrin+ and finally the populations of GFP+ cells from Pax7EGFP mice (green) or control mice (cyan) was displayed as histograms. (PDF 3145 kb) [file 13395_2018_169_MOESM5_ESM.pdf]

**Figure S5**

**a**

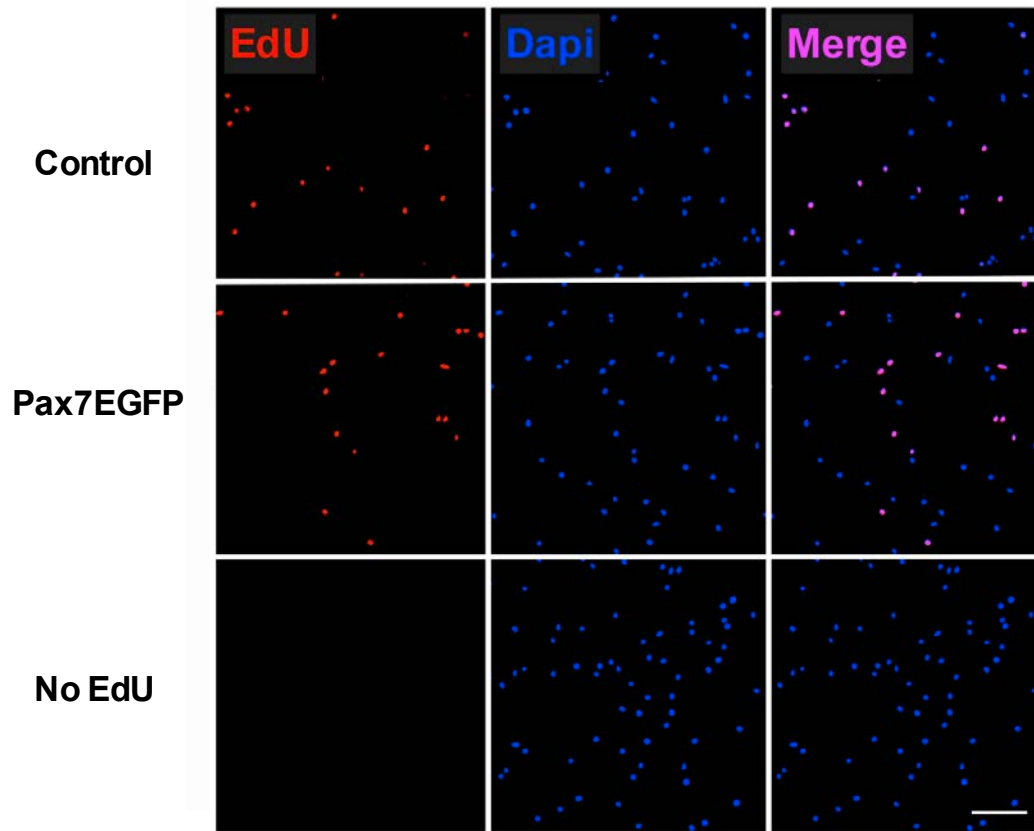

**b**

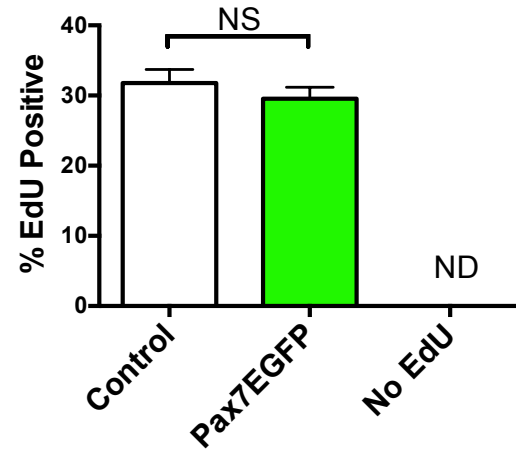

**c**

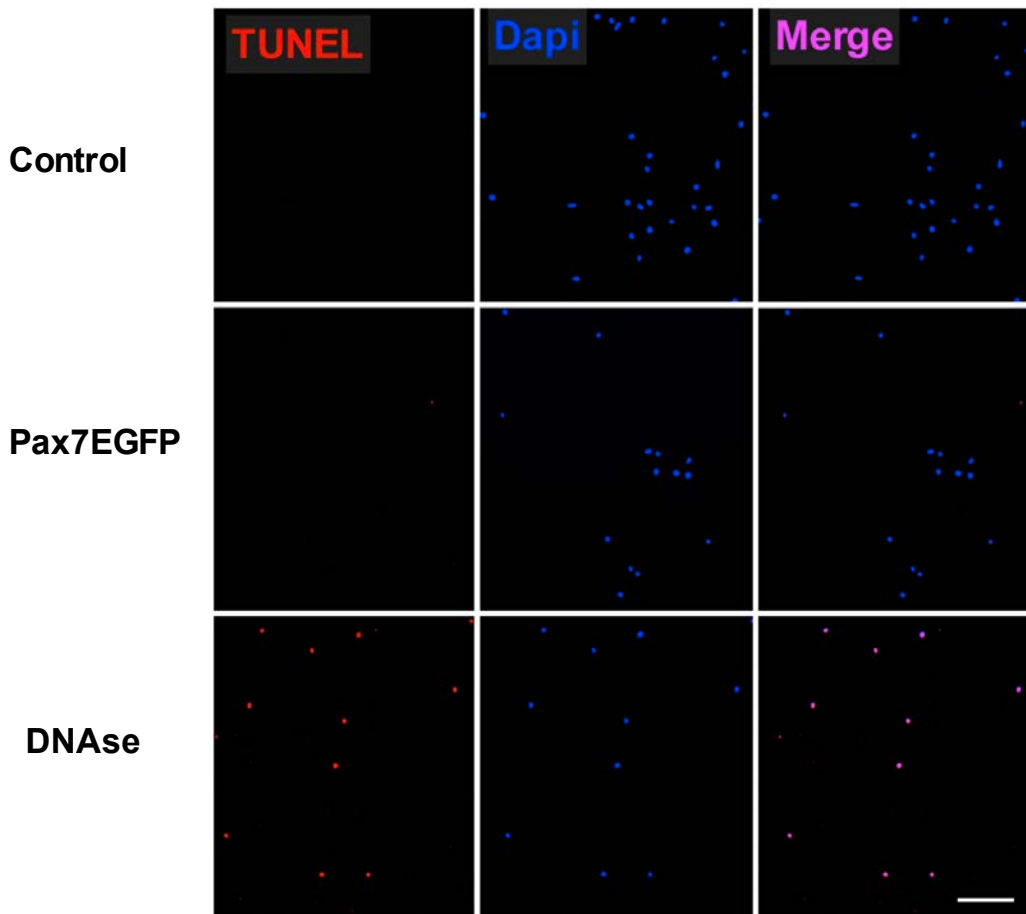

**d**

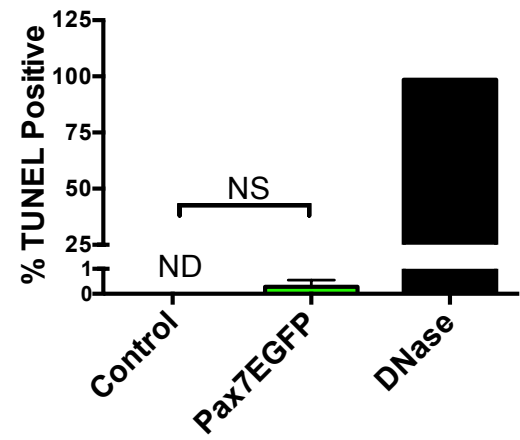

Supplement: Supplementary file 6 — Figure S5. Analysis of MuSC proliferation and cell death. (a) Measurement of proliferative capacity in MuSCs derived from control or Pax7EGFP mice. FACS-sorted MuSCs were plated on laminin-coated chamber slides in myoblast media containing bFGF for 2 days. EdU was added to the culture media, and cells were incubated for 2 h. Cells were fixed, and EdU incorporation was assayed by fluorescence microscopy. As a control, some cells were not treated with EdU. Scale bar = 100 μm. (b) Quantitation of data shown in (a). n ≥ 3 mice assayed, with at least N = 700 cells analyzed per group. (c) Measurement of cell death levels in FACS-sorted MuSCs by TUNEL assay. Cells were plated as in (a) and processed as described in the methods. As a positive control, fixed cells were treated with DNase. Scale bar = 100 μm (d) Quantitation of cell death levels shown in (c). ND, none detected. n ≥ 3 mice were analyzed; N > 400 cells were analyzed. (PDF 3145 kb) [file 13395_2018_169_MOESM6_ESM.pdf]

Figure S6

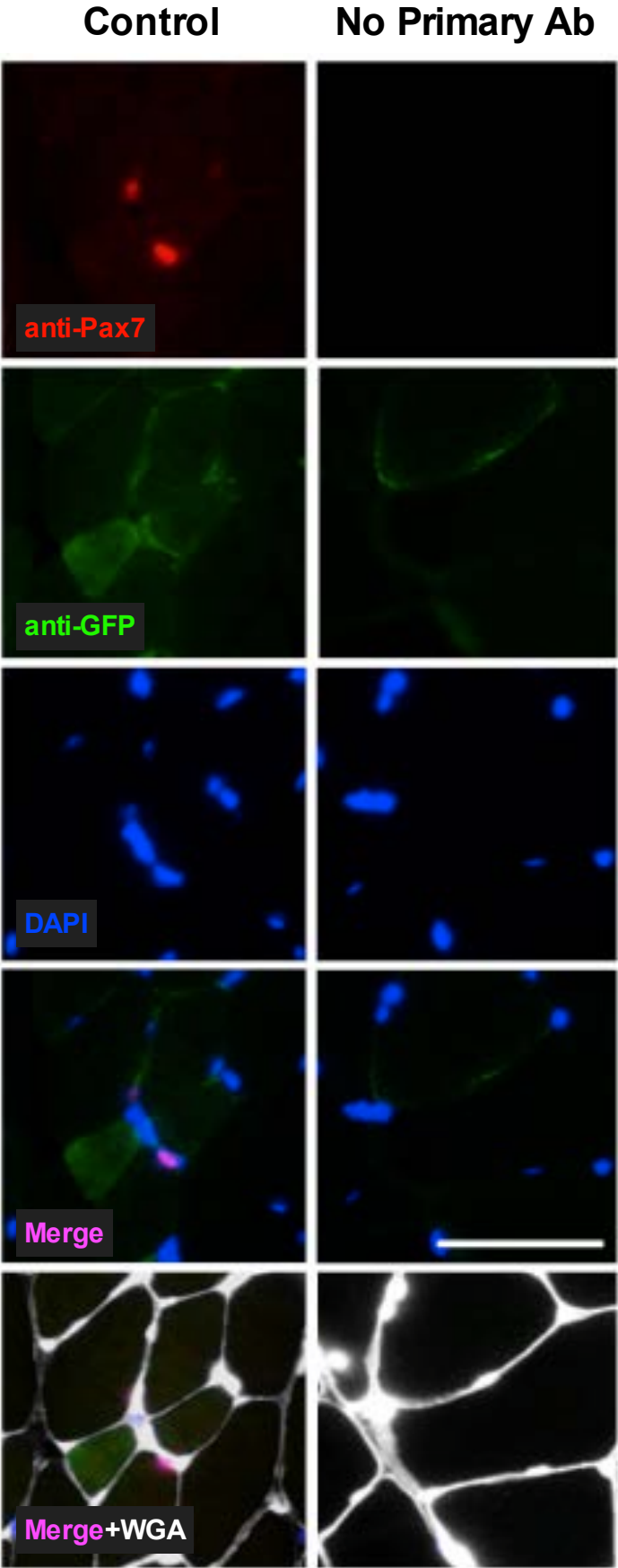

Supplement: Supplementary file 7 — Figure S6. Technical immunofluorescence control for Fig. 4b. (Left) Pax7EGFP negative mouse TA muscle cryosections were stained with Pax7 and GFP antibodies. (Right) TA muscle cryosections of Pax7EGFP mice processed the same as control muscle tissue, with the exception of no Pax7 or GFP antibody staining occurred. Note: while the GFP signal of Pax7EGFP mice survives our fixation protocol for cryosectioning, it does not survive EDTA-mediated antigen retrieval necessary for Pax7 staining. (PDF 89 kb) [file 13395_2018_169_MOESM7_ESM.pdf]

Figure S7

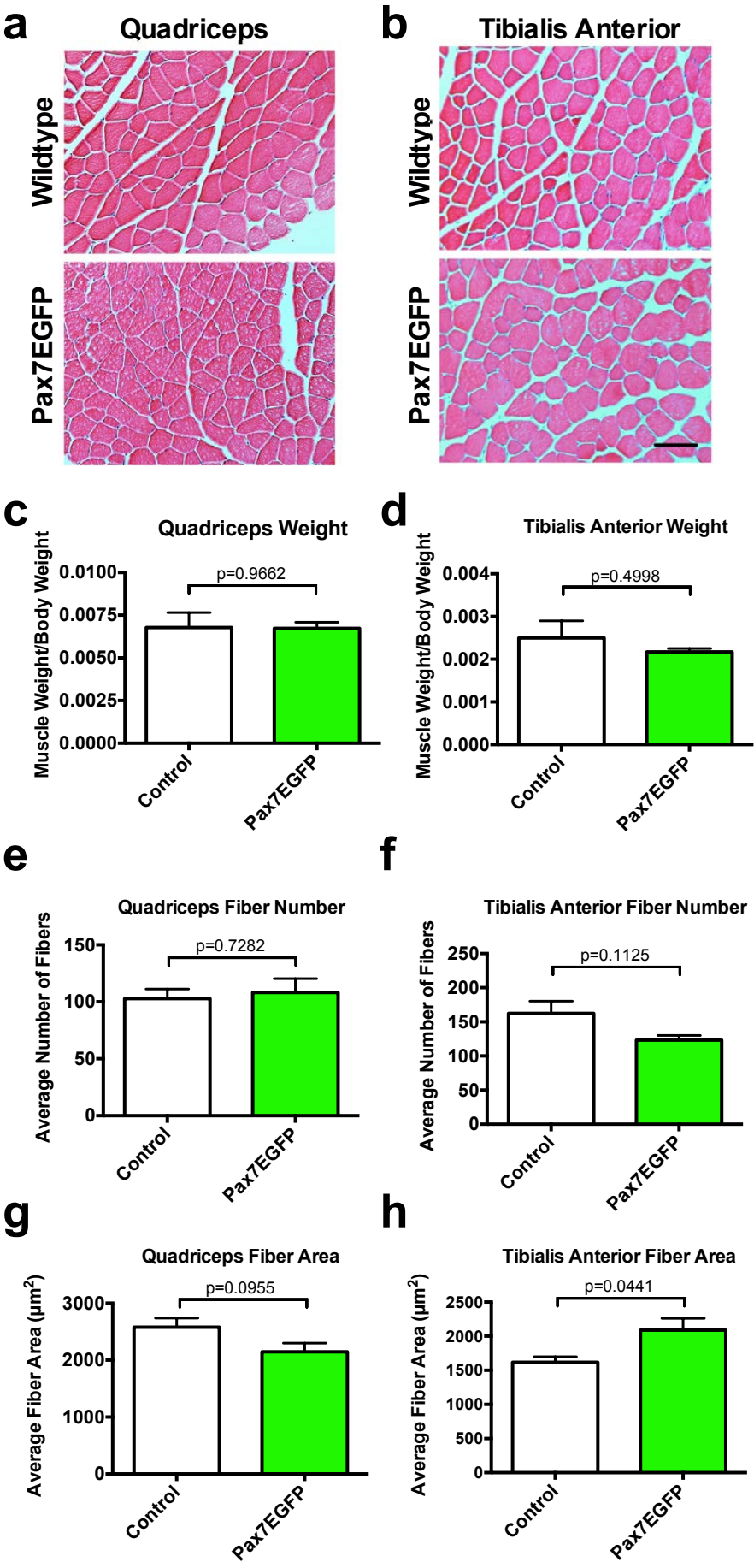

Supplement: Supplementary file 8 — Figure S7. Histological analysis of additional Pax7EGFP muscles. Representative images of quadriceps (a) and tibialis anterior (b) muscles from non-injured control (top) or Pax7EGFP heterozygous mice (bottom) stained with hematoxylin and eosin. (c–d) Individual muscle weights were normalized to total body weight per mouse. (e–f) Analysis of muscle fiber number. (g–h) Quantification of muscle fiber area. n = at least 3 mice (age and gender-matched) per genotype per condition. (PDF 180 kb) [file 13395_2018_169_MOESM8_ESM.pdf]

**Figure S8**

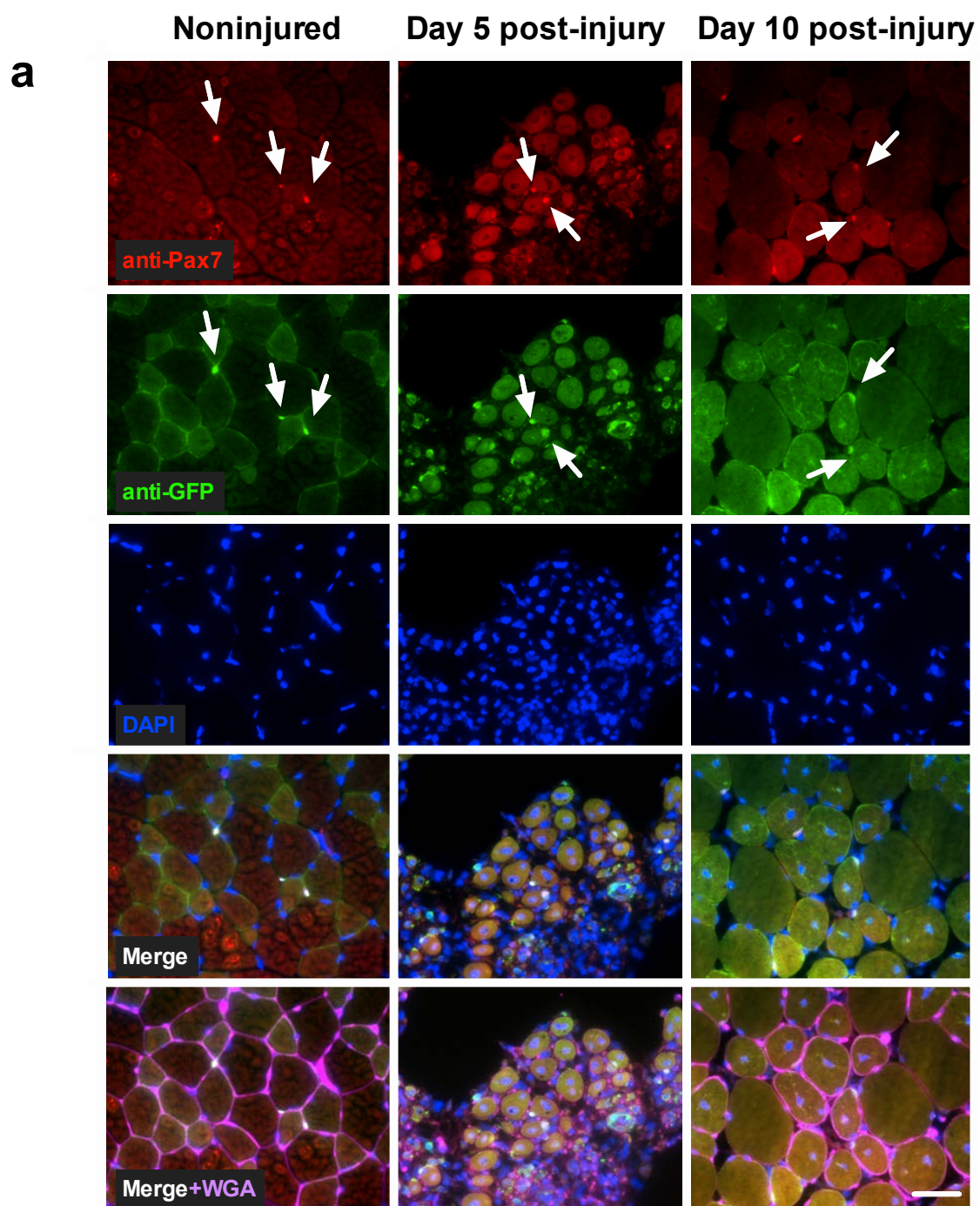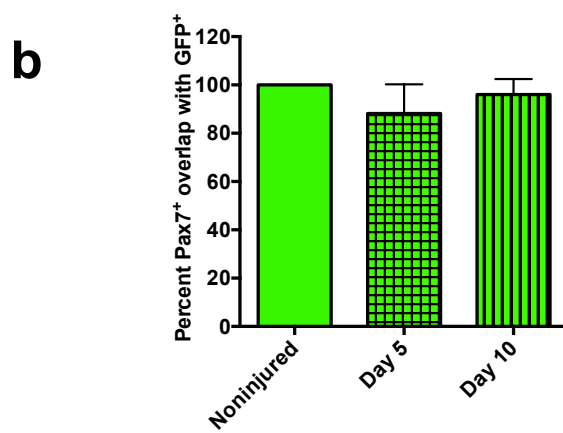

Supplement: Supplementary file 9 — Figure S8. Overlap of Pax7+ and GFP+ cells during regeneration. (a) Imaging of tibialis anterior cryosections stained with Pax7 and GFP under conditions of no injury, or 5 and 10 days post-injury. (b) Quantification of Pax7+ cells that overlap with GFP+ cells. n = 2–3 mice per condition. Displayed is the mean ± standard deviation. (PDF 605 kb) [file 13395_2018_169_MOESM9_ESM.pdf]
