# Supplementary material for: A robust Pax7EGFP mouse that enables the visualization of dynamic behaviors of muscle stem cells
Source: Skelet Muscle. 2018 Aug 24;8:27. doi: 10.1186/s13395-018-0169-7 (PMC6107960; doi:10.1186/s13395-018-0169-7)
Supplement: Supplementary file 4 — Supplementary methods [56, 58]. (DOCX 23 kb) [file 13395_2018_169_MOESM4_ESM.docx]

**Taqman Copy Number Variation Assay**

To extract DNA, tail snips were lysed in genomic DNA lysis buffer (100mM Tris, pH 8.0, 5mM EDTA, 200mM NaCl, 0.2% SDS, 0.2 mg/mL proteinase K) at 56ºC in a thermomixer (Eppendorf). The following day, DNA was precipitated with isopropanol, washed with 70% ethanol, and dried. DNA was resuspended in dH2O, and further purified using a Genomic DNA Clean and Concentrator-10 kit (Zymo), according to the manufacturer’s recommendations and eluted in 10 mM Tris, pH 8.0. DNA quality and concentration was assayed with a Nanodrop spectrophotometer. For the calibrator, known heterozygous Rosa^mTmG^ mice (Jackson stock 007676; backcrossed to a C57Bl/6 background in-house) were used (GFP copy number of 1). Reactions were set up in microamp fast optical 96-well reaction plates (Applied Biosystems), with each well receiving 1uL of 20X EGFP primers, 1uL of 20X Tfrc primers, 4uL of DNA (5ng/uL), 10uL of Taqman Universal PCR Master Mix (2X), and 4uL dH2O. All reactions were prepared in quadruplicate, including negative controls. Standard curves were generated from a dilution series of one sample, performed in duplicate. QPCR reactions were amplified using the Standard Curve method using a Quantstudio 6 Flex instrument (Applied Biosystems) with Quantstudio Real-Time PCR software v1.3. Primers used for the study were Fam-labeled EGFP primer sets (Mr00660654_cn; cat number 4400291) and Vic-labeled mouse Tfrc copy number reference assay primer sets (cat number 4458366). Following the reaction, the C_T_ threshold was set to 0.2 (or lower if needed to be in the linear range), with the autobaseline on. C_T_ values were imported into Copycaller software, and signals were normalized to the Rosa^mTmG^/Pax7Cre heterozygous mice. This method is useful for determining whether mice are homozygous or heterozygous, based on the number of GFP integrations into genomic DNA.

**Injured Muscle Histological and Immunofluorescence Staining**

Pax7EGFP heterozygous or control mice were injured with a single injection of the myotoxin, notexin (10μg/mL; 10μL; Latoxan) in the right Tibialis anterior muscle, as we previously described in [56, 58]. Muscles were harvested 5 or 10 days post-injury and were processed and imaged as described in the methods.

**MuSC In Vitro Proliferation and Cell Death Assays**

Pax7EGFP positive or control MuSCs were plated on laminin-coated 8-well chamber slides in myoblast media. For in vitro proliferation assays, MuSCs were plated for 2 days prior to the addition of EdU (Abcam, 10 μM final concentration) for two hours. MuSCs were fixed in 4% PFA/PBS and stained with the Click-iT EdU 594 Plus Kit (Life Technologies), according to the manufacturer’s instructions. For analysis of cell death, MuSCs were plated for 2 days, fixed in 4% PFA/PBS, and processed using the Click-iT TUNEL Alexa Fluor 594 kit (Life Technologies), according to the manufacturer’s instructions. As a positive control, fixed cells were treated with DNase I. Coverslips were mounted with Fluoromount G with DAPI. Cells were imaged on a Nikon eclipse 90*i* microscope equipped with Photometrics Coolsnap HQ2 14-bit digital camera.
